# Plasmon-Enhanced Antibacterial Activity of Chiral Gold Nanoparticles and In Vivo Therapeutic Effect

**DOI:** 10.3390/nano11061621

**Published:** 2021-06-21

**Authors:** Yuelong Xu, Hongxia Wang, Min Zhang, Jianhao Zhang, Wenjing Yan

**Affiliations:** National Center of Meat Quality and Safety Control, College of Food Science and Technology, Nanjing Agricultural University, Nanjing 210095, China; 2019108056@njau.edu.cn (Y.X.); whxia1010@163.com (H.W.); 17854338808@163.com (M.Z.); nau_zjh@njau.edu.cn (J.Z.)

**Keywords:** gold nanoparticles, chiral, d-cysteine, antibacterial activity, in vivo

## Abstract

d-cysteine (d-cys) has been demonstrated to possess an extraordinary antibacterial activity because of its unique steric configuration. However, inefficient antibacterial properties seriously hinder its wide applications. Here, cysteine-functionalized gold nanoparticles (d-/l-Au NPs) were prepared by loading d-/l-cysteine on the surface of gold nanoparticles for the effective inhibition of *Escherichia coli (E. coli)* in vitro and in vivo, and the effects on the intestinal microflora in mice were explored during the treatment of *E. coli* infection in the gut. We found that the antibacterial activity of d-/l-Au NPs was more than 2–3 times higher than pure d-cysteine, l-cysteine and Au NPs. Compared with l-Au NPs, d-Au NPs showed the stronger antibacterial activity, which was related to its unique steric configuration. Chiral Au NPs showed stronger destructive effects on cell membrane compared to other groups, which further leads to the leakage of the cytoplasm and bacterial cell death. The in vivo antibacterial experiment illustrated that d-Au NPs displayed impressive antibacterial activity in the treatment of *E. coli*-infected mice comparable to kanamycin, whereas they could not affect the balance of intestinal microflora. This work is of great significance in the development of an effective chiral antibacterial agent.

## 1. Introduction

Food safety is the main problem caused by foodborne pathogens, including *Escherichia coli* (*E. coli*), *Salmonella* and *Staphylococcus aureus* (*S. aureus*), etc. [1,2,3], which is the most concerning issue in public health [1,2,3]. *E. coli*, a Gram-negative, non-sporulating facultative anaerobe bacteria, is commonly found in the lower intestine and known as a harmless commensal of the gastrointestinal tract in warm-blooded animals [4,5,6]. However, *E. coli* can produce an enzyme called broad-spectrum β-lactamase that cause urinary tract infections. Furthermore, urinary tract infections may lead to some serious diseases that span from the gastrointestinal tract to extraintestinal sites such as the blood stream and central nervous system, which can be life-threatening [7,8]. Antibiotic treatment is currently the most effective method, but it is easy to promote the emergence of multi-drug resistant *E. coli*, which constitutes one of the dominant challenges to human health [9,10,11]. Additionally, the obvious and continuous decrease in the number of approved antibiotics in the past 10 years has exacerbated the increasingly threatening circumstances [12,13]. This situation has created an urgent need to discover novel antibacterials and treatment strategies [14].

Inorganic nano antibacterial agents have become a research hotspot in recent years due to their good heat resistance, broad-spectrum antibacterial properties and no drug resistance [15,16,17,18,19]. Among them, silver nanoparticles (Ag NPs) were once thought to be the most promising alternative to antibiotics and were widely used in many different fields [20,21]. However, they have been shown to have obvious cytotoxicity and genotoxicity (i.e., low selectivity) against healthy mammalian cells, which severely limits their practical application [22]. Other inorganic nanomaterials, for example, titanium dioxide (TiO_2_) NPs and graphene oxide (GO), cause cytotoxicity against normal mammalian cells because of the excess reactive oxygen species (ROS) [23]. Thus, developing new nanomaterials with targeted antibacterial activity against bacteria represents a great challenge.

Gold nanoparticles (Au NPs) have received more attention due to their excellent biocompatibility, stability and low cytotoxicity [24,25,26,27,28]. However, the antibacterial activity of Au NPs cannot adequately meet the needs of practical applications compared with Ag NPs and TiO_2_ NPs [29,30,31]. It has been demonstrated that a modification of the surface with an antibiotic can effectively enhance the antibacterial activity of gold nanoparticles [32], whereas the process often requires exogenous antimicrobial laser irradiation and still might not be able to avoid the emergence of drug resistance. Fortunately, the high binding affinity of gold atoms facilitates the conjugation of other functional ligands onto the surface of Au NPs, such as specific proteins and amino acids [33,34], which provides the possibility for gold nanoparticles to be exploited as antibacterial agents without existing antibiotics [35].

d-cysteine (d-cys), an enantiomer of natural l-cysteine (l-cys), has been proved to significantly inhibit the growth of Gram negative *E. coli* by inhibiting the activity of threonine deaminase, which may further affect the synthesis of isoleucine, leucine and valine in bacteria [36,37,38]. In spite of this, d-cys is not widely used in practical applications because of its inefficient antibacterial properties. Here, functional gold nanomaterials (d-/l-Au NPs) with stable antibacterial effects were synthesized using cysteine as a ligand. The structural characterization of new materials and the antibacterial activity against *E. coli* were investigated in vitro. To investigate the antibacterial mechanism of d-/l-Au NPs against *E. coli*, confocal laser scanning microscopy (CLSM), scanning electron microscopy (SEM) and transmission electron microscopy (TEM) were performed. Moreover, the therapeutic effect of d-Au NPs on intestinally infected mice and the influence on intestinal flora were investigated, compared with kanamycin, which further demonstrates the prominent bactericidal application potential. The work we mentioned above has never been reported before, and it has important theoretical significance for the development of a new chiral antibacterial agent.

## 2. Materials and Methods

### 2.1. Materials

d-cysteine, l-cysteine, sodium citrate, gold tetrachloroaurate trihydrate (HAuCl4), kanamycin and sodium hydroxide were purchased from Sigma-Aldrich (Shanghai, China). Broth edium and eosin-methylene blue agar (EMB) were purchased from Hope Bio-technology Ltd. (Qingdao, China). LIVE/DEAD^®^BacLightTM Bacterial Visibility Kit was purchased from Invitrogen. *Escherichia coli* (*E. coli*) was obtained from Guangdong Huankai Microbiology technology co. Ltd. Guangdong province, China)

### 2.2. Synthesis of Chiral Cysteine Functionalized Gold Nanoparticles

Chiral cysteine functionalized gold nanoparticles (d-/l-Au NPs) were prepared by the following method: first, 2.5 mL of 12 mM HAuCl_4_ was brought to boil in 50 mL of distilled water. Then, 1.0 mL of 1% trisodium citrate was quickly added to the sample under high-speed stirring. The solution was kept for 20 min until the color turned wine red. Finally,1.0 mL of 0.6% NaOH and 1.0 mL of 1.8% cysteine was in turn added to the solution under vigorous stirring for 2 h. The solution was centrifuged at 11,000 rpm for 20 min, and the precipitate was re-suspended in 10 mL distilled water to obtain experimental materials.

### 2.3. Bacterial Culture

*E. coli* (ATCC25922) were cultured in LB medium at 37 °C for 12 h under shaking at 200 rpm. The bacterial suspension was centrifuged at 6000 rpm for 10 min at 4 °C and re-suspended in sterile saline solution (0.9% NaCl). The concentration of *E. coli* cells was quantified to 10^7^colony-forming units per millimeter (CFU/mL) by OD _600_ measurements.

### 2.4. Antibacterial Activity Assays

To measure the dynamic bacteriostatic properties of d-/l-Au NPs on *E. coli*,1 mL of 10^7^ CFU/mL *E. coli* suspension was mixed with1 mL of 3 mg/mL d-Au NPs or l-Au NPs solution, respectively; the samples were cultured at 37 °C for 24 h. The aseptic saline group was used as negative control; Au NPs, l-cys and d-cys with the same content were used as positive control, respectively. The bacterial growth was evaluated every 1 h by recording the optical density at 600 nm (OD_600_ nm) [39,40].

The antibacterial activity of d-/l-Au NPs (3 mg/mL) against *E. coli* (10^7^ CFU/mL) after 6 h was determined by plate counting method. The mixture of D-/l-Au NPs solution and *E. coli* suspension was collected and the 10-fold serially diluted cells were spotted onto Violet Red Bile Agar (VRBA). The CFUs were counted after overnight incubation at 37 °C. The percentage of dead bacteria was calculated according to the previous article [41].

### 2.5. Live/Dead Bacterial Assay

Confocal laser scanning microscopy (CLS) was performed to characterize the destruction of D-/L-Au NPs on the cell membrane of *E. coli*. d-/l-Au NPs were incubated with 10^7^ CFU/mL bacterial suspension at 37 °C for 24 h [42]. The solution was then mixed with dye solution containing SYTO9 and propidium iodide (PI) for 20 min at room temperature. The stained sample was observed under a confocal laser scanning microscope (TCS-SP2, Leica, Weztlar, Germany). PI fluorescent dye can only bind to the DNA in the cell membrane of incomplete bacteria, emitting red fluorescence. The SYTO9 fluorescent dye can bind to DNA in the cell membrane of intact bacteria, producing green fluorescence. The effect of d-/l-Au NPs on the cell membrane of *E. coli* was evaluated by the ratio of red/green fluorescence, which can be used to indicate the ratio of live and dead bacteria.

### 2.6. In Vivo Antibacterial Activity in Infected Mice

The animals were maintained in the Center for Experimental Animals at Nanjing Agricultural University (permit number: SYXK (SU) 2017-0007), following the protocols approved by the Ethical Committee of Nanjing Agricultural University, China. Twenty female BALB/c mice (6–8 weeks, 18–22 g) were purchased from the Center of Comparative Medicine Centre of Yangzhou University (permit number: SCXK (SU) 2012-0004) and allowed to acclimatize for 3 days in the laboratory. A bacteria-infected mouse model was created by oral gavage with 200 μL of *E. coli* containing 1 × 10^7^ CFU/mL into female BALB/c mice (n = 15) [43]. The healthy mice (n = 5), as a group, were not infected with *E. coli*. One day postinfection with *E. coli*, mice were randomly divided into three groups (n = 5) and treated with 200 μL PBS, kanamycin (2.5 mg kg^−1^) and d-Au NPs (2.5 mg kg^−1^), respectively, twice in one day with a 12 h interval. The activity and body weight of mice were recorded throughout the entire treatment period. After three days, all mice were sacrificed and the intestinal tissues (colon, cecum and small intestinal) were isolated, which were then placed in sterile PBS. Aliquots of diluted homogenized intestinal tissues were plated on agar, on which the grown colonies were counted for analysis [44]. The number of *E. coli* in the intestinal tissues of mice was evaluated by the LB-agar plate dilution method.

### 2.7. Changes of Intestinal Microflora in Mice

To explore the effect of d-cysteine-functionalized Au NPs on the changes of intestinal microflora in mice, fecal genomic DNA of mice was extracted by QiAamp DNA stool MiniKit (NO.51504, Qiagen, Venlo, Germany) [45,46]. Total DNA was quantified using a NanoDrop 2000^®^ spectrophotometer (Thermo Fisher Scientific, Massachusetts, USA). The resulting DNA was amplified with barcoded specific bacterial primers targeting the 16S rRNA gene V3 and V4 region using primers with the Primer F (Illumina adapter sequence 1+CCTACGGGNGGCWGCAG) and Primer R (Illumina adapter sequence 2+GACTACHVGGGTATCTAATCC). The Library quality was determined using an Agilent 2100 Bioanalyzer (Agilent Technologies, California, USA). A unit of 250 bps paired-end sequencing of the amplicon was performed using MiSeq Reagent Kit v3 (Illumina, California, USA). After splicing, quality control and filtering the Barcode sequence, the high-quality clean reads were obtained for OTUs cluster analysis. The OTU representation sequence was compared with the Geen Gene database. According to the clustering results, Alpha and Beta diversity analyses were performed. The kinds and abundance of the intestinal microflora were analyzed at the level of phylum and family.

## 3. Results and Discussion

### 3.1. Characterization

**Figure 1:**
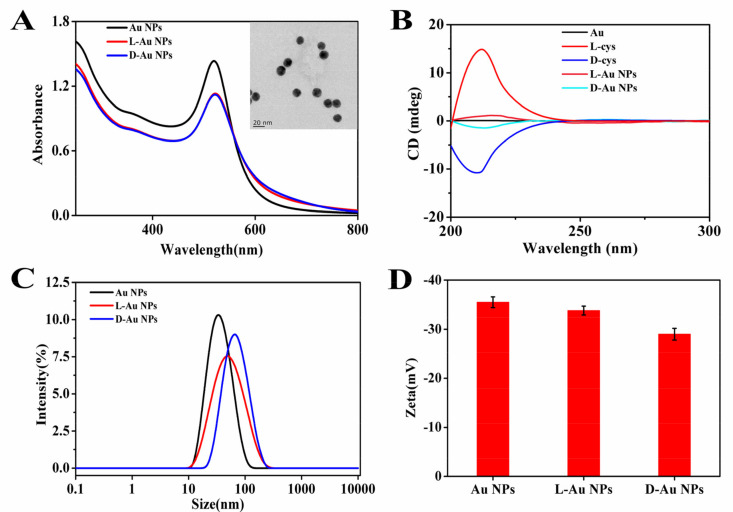
Characterizations of cysteine-functionalized gold nanoparticles(d-/l-Au NPs). (**A**) Absorption spectrum of Au NPs, l-Au NPs and d-Au NPs (Insert: TEM image of d-Au NPs). (**B**) Circular dichroic spectrum, (**C**) particle size distribution and (**D**) zeta potential of Au NPs and chiral Au NPs. (Dates are presented as the mean ± s.d.).

Transmission electron microscopy (TEM) was used to characterize the morphology of chiral Au NPs. As showed in the Figure 1A insert, chiral Au NPs were well-dispersed with the diameter of 15 ± 2 nm, which was not significantly different from spherical Au NPs. The UV-VIS spectrum of d-/l-Au NPs compared with Au NPs in the absorption maximum shifts from 520 to 522 nm (Figure 1A). Circular dichroism (CD) was used to determine the stereo configuration of chiral Au NPs [47]. As shown in Figure 1B, l-Au NPs and d-Au NPs had an opposite characteristic peak in the wavelength range of 20–250 nm, which were consistent with L-cysteine and d-cysteine, respectively. As pure Au NPs showed no characteristic peaks in the ultraviolet region, it can be indicated that l-/d-cysteine were successfully modified on the surface of Au NPs (Figure 1B). To explore the effect of chiral cysteine on the surface of gold nanoparticles, the dynamic light scattering (DLS) and Zeta potential were measured. DLS results showed that the mean hydrodynamic sizes of Au NPs, l-Au NPs and d-Au NPs were 32.67, 50.75 and 62.06 nm, respectively. The diameters of NPs were slightly larger than the size obtained in TEM images, which may be related to the hydration of chiral Au NPs by the surrounding water (Figure 1C) [48]. The Zeta potential values of Au NPs, L-Au NPs and d-Au NPs were −35.5 ± 1.1, −33.8 ± 0.9 and −29.0 ± 1.2 mV, respectively, demonstrating that the coupling of SH can reduce the negative charge on the surface of Au NPs (Figure 1D).

### 3.2. In Vitro Antibacterial Activities of Chiral Au NPs

**Figure 2:**
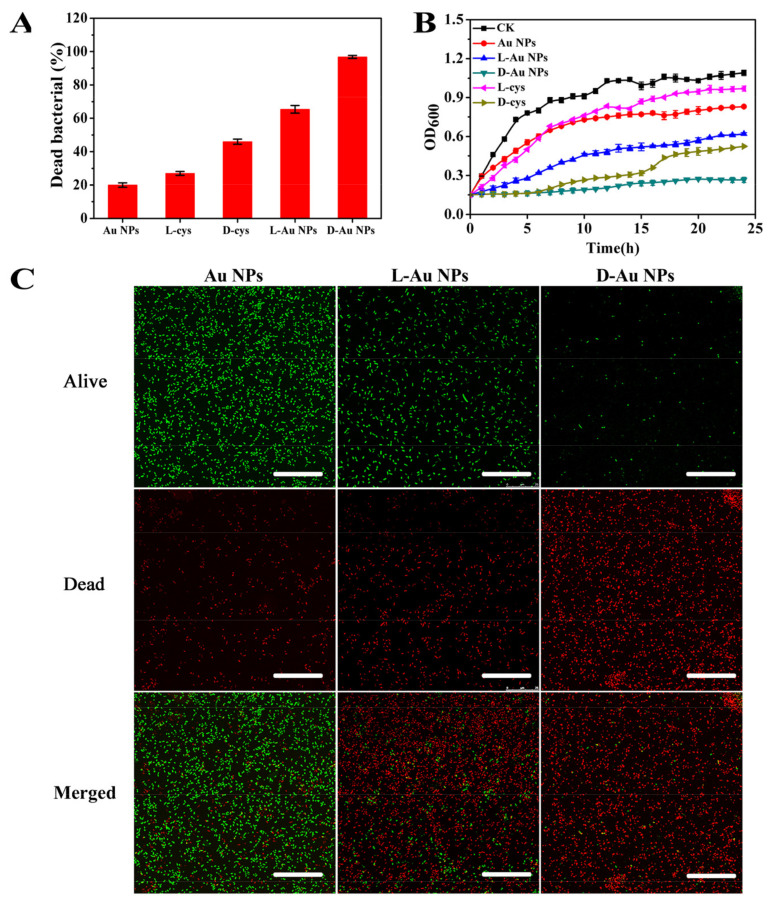
(**A**) Percentage of dead *E. coli* after treatment with d-/l-cysteine, Au NPs and d-/l-Au NPs at 3 mg/mL for 12 h, respectively. Insert: Agar plates showing bacterial colonies after different treatments (Dates are presented as the mean ± s.d.). (**B**) The growth kinetics of *E. coli* incubated with d -/ l -cysteine, Au NPs and d-/l-Au NPs for 24 h, respectively. (**C**) Confocal microscopy images of *E. coli* after treatment with Au NPs, l-Au NPs and d-Au NPs, for 12 h, respectively. The scale bar is 25 μm.

To evaluate the antibacterial activity of d-/l-Au NPs, *E. coli* as a typical Gram-negative bacteria was used in the test. Agar plate images and quantitative histograms of dead bacterial are shown in Figure 2A. It can be seen that cysteine-functionalized Au NPs displayed remarkable bactericidal properties against *E. coli* compared with free d-/l-cysteine or pure Au NPs at the same dosage. The lethality rate of d-Au NPs and l-Au NPs toward *E. coli* were increased to 96.8% and 65.4%, respectively, while the lethality rate of d-cys, l-cys or Au NPs was only 46%, 27% and 20%, respectively. The significant antibacterial activity of d-/l-Au NPs was attributed to two aspects: first, the stability of cysteine is greatly increased after immobilization via Au-S bonding on the surface of Au NPs, which has a significant association with antimicrobial properties [49]. Besides, Au NPs may concentrate antibacterial cysteine on their surfaces to result in polyvalent effects; thus, d-/l-Au NPs can specifically attack biological targets in bacteria [50,51]. We also noticed that d-Au NPs displayed higher antibacterial activity than l-Au NPs, which may be related to their unique stereo configuration. D-amino acids have been shown to play an important role in the regulation of bacterial physiological structure and function [52].

To further demonstrate the antibacterial property of chiral Au NPs, the growth curve of *E. coli* cells after treatment was measured at OD _600_ nm within 24 h. As shown in Figure 2B, *E. coli* treated with Au NPs and l-cys showed a delayed exponential growth compared with the control group (CK), but the OD value increased rapidly after 4 h. The d-cys and l-Au NPs solutions were discovered to moderately inhibit bacterial growth and showed better antimicrobial activity than the pure l-cys and Au NPs. In contrast, a slight increase was observed in the OD value for bacteria treated with d-Au NPs within 24 h, showing the strongest and long-term bactericidal activity as opposed to the other samples [53]. Moreover, in vitro antibacterial activity of d-Au NPs was evaluated using the Live/Dead bacterial viability assay, which can be used to distinguish between live cells with intact membranes and dead cells with damage membranes. As shown in Figure 2C, almost all *E. coli* treated with d-Au NPs displayed red fluorescence; this means that most bacteria after treatment were dead and the red fluorescence intensity was stronger compared with Au NPs or l-Au NPs, indicating that d-Au NPs exhibited high antibacterial activity against *E. coli*. The result is consistent with that determined by the plate count method.

### 3.3. Antibacterial Mechanism of d-/l-Au NPs

**Figure 3:**
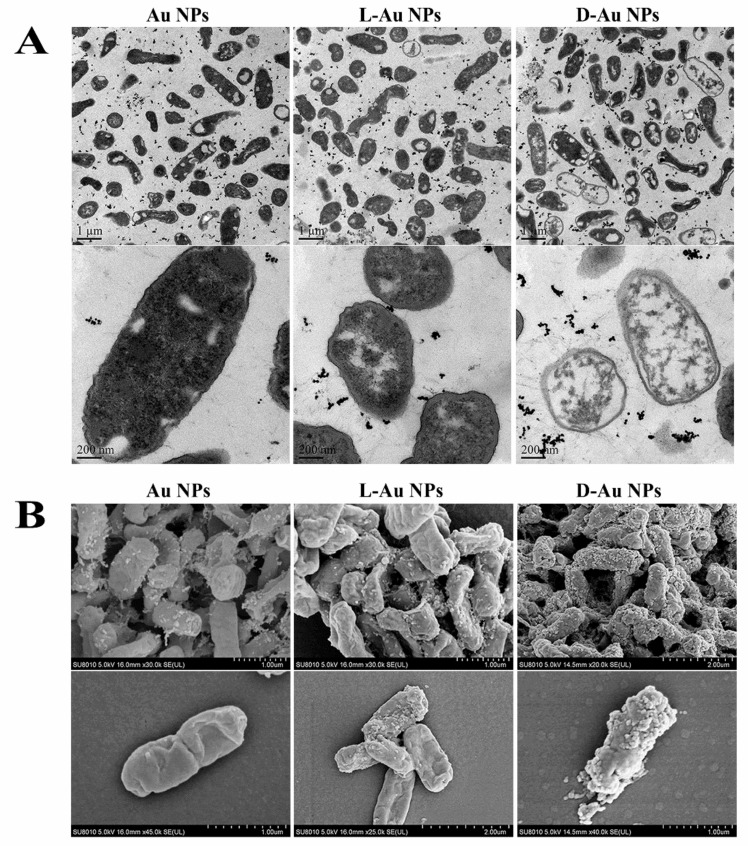
(**A**) TEM and (**B**) SEM images (5.0 kV for work) of *E. coli* after treatment with Au NPs, l-Au NPs and d-Au NPs for 12 h, respectively.

To gain insight into the antimicrobial mechanism of d-/l-Au NPs, 3 mg/mL of nanomaterials were incubated with 10^7^ CFU/mL of *E. coli* for 12 h; the morphology changes of bacteria were then investigated by transmission electron microscope (TEM) and scanning electron microscope (SEM). As shown in Figure 3A, compared with Au NPs or l-Au NPs, d-Au NPs seriously damaged the cell membrane of *E. coli*, led to cytoplasm leakage and cell death. The results can also be proved by SEM images. It is clear that compared with Au NPs and l-Au NPs, *E. coli* displayed more severe changes in morphology in the presence of the d-Au NPs; the wrinkled cell wall and the disappeared cellular integrity may reflect leakage of cytoplasmic content outside the bacterial cell (Figure 3B). This is the leaked cytoplasm (proteins and DNA) from bacteria adhered to the surface of d-Au NPs, resulting in the formation of some tiny particles outside the cell membrane [54].

The severe disruption of d-Au NPs was attributed to the inherent antibacterial property of d-cys and the synergistic effects of Au NPs [41]. We noticed that nanoparticles were mainly adsorbed on the cell membrane or aggregated outside the cell, which may be related to the long treatment time. According to the previous report, engineered nanomaterials enter cells via several pathways, including nonspecific endocytosis, physical electroporation or targeted uptake based on the surface functionalization [55]. As for the antibacterial mechanism of d-Au NPs, we hypothesis that d-cys-modified Au NPs may specifically bind to the proteins on the cell membrane of bacteria and result in the destruction of cell membrane integrity by changing the structure of proteins [56], which was consistent with the TEM images (Figure 3A). Chiral nanomaterials-induced structure changes of proteins have been reported in previous works [57,58]. On the other hand, a portion of d-Au NPs may enter the bacteria by endocytosis, affecting the physiological activity of bacteria through interactions with proteins and DNA, resulting in cell death. Gong et al. reported that D-glutamic acid modified graphene quantum dot (GQD) can specifically bind to cytoplasmic MurD and inhibit its catalyzing activity, which was directly related to the biosynthesis of intracellular peptidoglycan and the damage of cell wall [59]. It has been reported that d-cys can inhibit the catalyzing activity of threonine deaminase in *E. coli* and further affect the synthesis of essential amino acids in bacteria. The antibacterial mechanism of d-Au NPs at the molecular level will be systematically investigated through future research.

### 3.4. In Vivo Therapy of Mice Suffering from E. coli Infection

**Figure 4:**
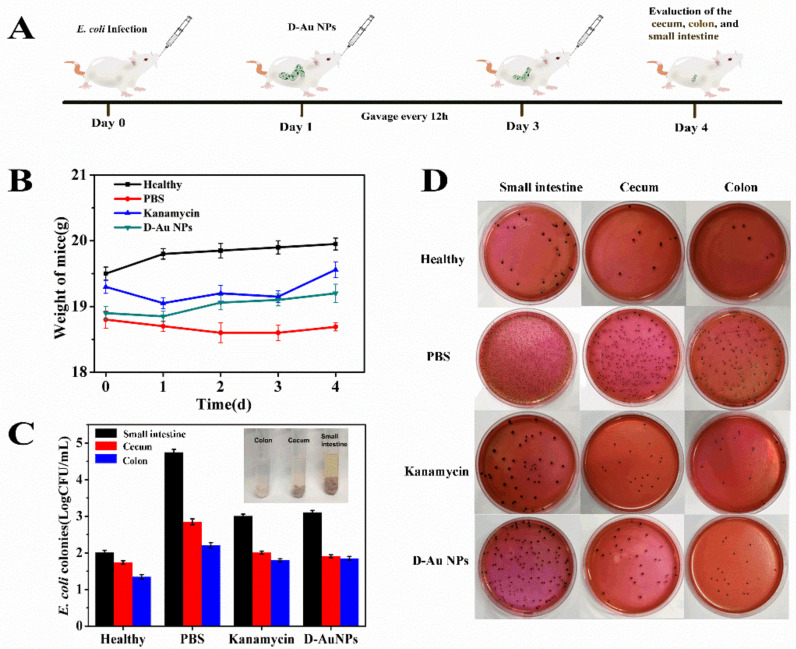
(**A**) Schematic diagram of the in vivo therapy of *E. coli* infection with d-Au NPs. Mice were evaluated after 3-days of gavage with PBS, d-Au NPs and kanamycin, respectively. (**B**) Changes in body weight in mice during the treatment. (**C**) The survived *E. coli* in the intestine of the infected mice after different treatments. (Insert: The colon, cecum and small intestine harvested from the infected mice after treatment with D-Au NPs). (Dates are presented as the mean ± s.d.). (**D**) Agar plates showing bacterial colonies of the intestine after different treatments.

To assess antibacterial efficacy in a complex physiological environment, the mice suffering from *E. coli* infection in the intestine were treated with d-Au NPs (Figure 4A). The healthy group was not infected with *E. coli*. Mice infected with *E. coli* in the model group were treated with PBS. The positive control was treated with kanamycin, which was used for clinical therapy for most Gram-negative bacteria infections. Changes in body weight in mice during treatment are important indicators to assess therapeutic efficacy. As shown in Figure 4B, the body weight of mice treated with d-Au NPs showed a slight decrease on the first day and began to slowly recover and increasee at 2 days postinfection; in contrast, the body weight of mice treated with kanamycin recovered after 4 days postinfection, and the body weight of mice treated with PBS did not significantly increase. Figure 4C shows the bacterial CFU in the colon, cecum and small intestine in mice after treatment. The results indicated that treatment with d-Au NPs on *E. coli* infected mice resulted in similar kanamycin effects. Specifically, after treatment with d-Au NPs, the *E. coli* count in small-intestine samples was lower by more than 1.5 orders of magnitude compared to the PBS-treated groups, similar to the kanamycin group, and the bacterial concentrations in the colon and cecum in mice were down to the level close to the healthy group (Figure 4D). d-Au NPs are effective in the treatment of *E. coli* infection in mice.

### 3.5. Effects on the Structure of Intestinal Microflora in Mice

**Figure 5:**
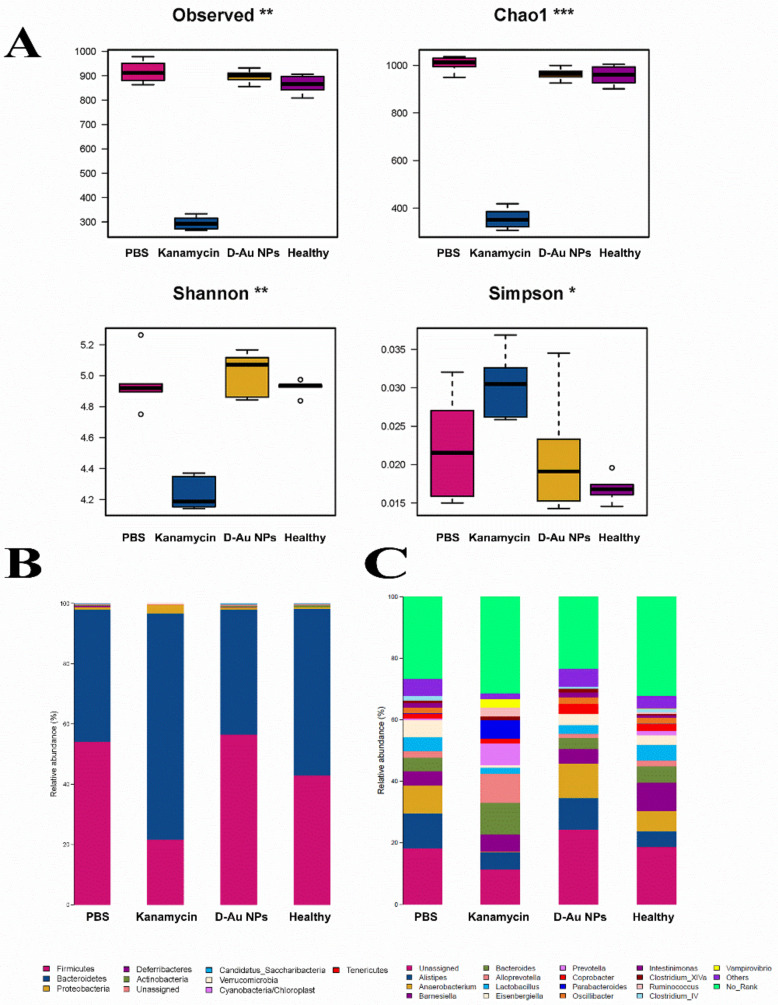
(**A**) Box plot of Alpha diversity index between PBS, kanamycin, d-Au NPs and healthy groups. (**B**) Histogram of relative abundance of species at the phylum level. (**C**) Histogram of relative abundance of species at the genus level. Dates are presented as the mean ± s.d. *: The mean value of the relative abundance of the species in the group of samples, *** > ** > *.

The balance of intestinal microflora is closely related to chronic metabolic diseases, including diabetes, hypoglycemia and gout, which is a matter of great concern in the treatment of infections in the gut. Here, the effect of d-Au NPs on the diversity of microflora in the treatment of *E. coli* infection in mice was explored [60]. Figure 5A displays the α-diversity of intestinal microflora in mice after a 3-day treatment with d-Au NPs, kanamycin (positive control) and PBS (negative control), respectively, containing richness (Chao1 index) and evenness (Shannon and Simpson indexes). The Chao1, Shannon and Simpson indexes showed no obvious difference between the d-Au NPs treated groups and the healthy group. In contrast, the richness of microflora severely decreased after treatment with kanamycin, the Chao1 index decreased from 957.83 to 355.77, and the Shannon index decreased from 4.92 to 4.23. The results indicated that oral antibiotics for 3 days significantly decreased the diversity of intestinal microflora in mice, whereas d-Au NPs cannot affect the richness and evenness of intestinal microflora [61].

We further investigated the relative abundance of Firmicutes, Bacteroidetes and Proteobacteria in microflora changed after a 3-day administration of d-Au NPs [62]. As shown in Figure 5B, d-Au NPs slightly changed the abundance of Firmicutes, Bacteroidetes and Proteobacteriain microflora in mice compared with the healthy group, whereas kanamycin greatly changed the abundance of bacteria on the phylum level. Kanamycin effectively killed the negative bacteria in the gut so that the proportion of Gram-positive bacteria increased, for example, as in Proteobacteria. At the genus level, d-Au NPs could slightly increase the abundance of Anaerobacterium, and decrease the abundance of Barnesiella, Bacteroides and Lactobacillus. In contrast, kanamycin treatment could greatly increase the relative abundances of Alloprobacillus, Parabacteroides and Vampirovibrioover levels of the healthy group. These results demonstrated that d-Au NPs treatment could not disturb the balance of intestinal microflora during the treatment of infections in the gut.

## 4. Conclusions

In summary, we synthesized an efficient chiral antimicrobial agent by loading d-/l-cysteine on the surface of Au NPs (d-/l-Au NPs). In vitro experiments demonstrated d-Au NPs exhibited the strongest antibacterial activity with *E. coli* compared with other groups, owing to the synergistic effect of Au NPs and d-cysteine. Mechanism research indicated that the strong antibacterial ability of d-Au NPs can be attributed to damaging cell integrity, which may be caused by a number of reasons. An in-depth investigation of antibacterial mechanisms will be performed in following research. Most importantly, in vivo experiments demonstrated that d-Au NPs displayed a therapeutic effect on the intestinally infected mice comparable with kanamycin, and they could not cause disturbances in intestinal microflora during the treatment of infections in the gut. This research offers a novel approach for the rational design of an effective antibacterial nano agent for the therapy of bacterial infection.

## Data Availability

Not applicable.
